# Do conscientious employees have a high level of work engagement? The roles of presenteeism and perceived organizational support

**DOI:** 10.3389/fpsyg.2024.1485025

**Published:** 2024-12-18

**Authors:** Hui Sun, Shuai Wang, Wei Zhang, Ling Sun

**Affiliations:** ^1^School of Business, Jiangsu Ocean University, Lianyungang, China; ^2^School of Economics and Management, Wenzhou University of Technology, Wenzhou, China; ^3^School of Environmental Engineering, Xuzhou Institute of Technology, Xuzhou, China

**Keywords:** conscientious employees, work engagement, presenteeism, perceived organizational support, conscientiousness

## Abstract

In recent years, work engagement garnered significant attention from both the business community and academia. Drawing on conservation of resources (COR) theory, this study investigates the mechanisms and boundary conditions through which conscientiousness influences work engagement. Through an empirical survey of 376 employees, the study found that, first, conscientiousness positively predicts employees’ work engagement; second, presenteeism partially mediates the relationship between conscientiousness and work engagement; third, perceived organizational support (POS) negatively moderates the relationship between conscientiousness and presenteeism while positively moderating the relationship between presenteeism and work engagement; fourth, POS moderates the indirect effect of conscientiousness on work engagement via presenteeism, whereas the mediated relationship is weakened when employees exhibit a higher POS. These findings advance our theoretical and practical knowledge of how personality traits and situational factors jointly affect employees’ work engagement, providing empirical data for a dialectical perspective on conscientious employees and enhancing their work engagement.

## 1 Introduction

Work engagement is a hot topic for researchers and managers, as it is a crucial indicator of employee enthusiasm and involvement at work (Malinowska and Tokarz, 2020). Particularly, as the market competition environment becomes increasingly dynamic and blurred, as well as the rapid changes in information technology, the question of how to keep employees passionate and energetic, maintaining high levels of work engagement requires systematic and sustained consideration.

As a crucial component of the Big Five personality traits, conscientiousness is a personal resource that may predict employees’ proactive organizational behavior and serve as an important factor in stimulating individual work engagement (Tu et al., 2020). Conscientiousness has always been regarded as a highly respected personality trait in traditional business management. Therefore, academics conducted extensive research on its positive impacts, including higher subjective well-being among employees (Anglim et al., 2020), higher job satisfaction (Huo and Jiang, 2021), fewer work-related accidents (Postlethwaite et al., 2009), a lower likelihood of ostracizing the workplace (Rudert et al., 2020), and higher LMX quality (Lapierre and Hackett, 2007). Do conscientious employees experience high work engagement?

Tu et al. (2020) indicated that conscientious employees are more inclined to exert effort toward their organization. Harshleen Kaur Sethi and Barclays Shared Services Pvt. Ltd. (2017) suggested that conscientiousness positively influences work engagement. However, recent research in personality theory introduced the “too-much-of-a-good-thing effect” in the relationship between conscientiousness and ideal outcomes, challenging the “more is better” view that has been dominating research on this trait for a long time (Dupré et al., 2022). Consistent with this, some studies indicate that conscientiousness also has a “dark side,” which can have negative impacts on employees, primarily manifested in lower life satisfaction (Boyce et al., 2010) greater work stress (Lin et al., 2015), and greater performance pressure (Liu et al., 2022).

A review of the relevant literature identified two research gaps. First, previous studies mostly emphasized the positive effects of conscientiousness on organizations and employees, with little research on the negative impacts of this trait (Liu et al., 2022). Presenteeism can also be defined as working while sick (Johns, 2010). The culture in China emphasizes values such as diligence, hard work, and perseverance, presenteeism is considered an important virtue (Chen et al., 2021). Recent research examined the prevalence of presenteeism, revealing that this attendance behavior is widespread among employees (Côté et al., 2021). Presenteeism can have numerous negative impacts on organizations (Côté et al., 2021), such as productivity losses, leading to more absences (Johns, 2011). In Chinese society, where hard work is highly valued and overtime is prevalent, employee presenteeism may be more common, drawing widespread attention from scholars of organizational behavior, human resources, organizational psychology, and health psychology (Li et al., 2020). Clarifying the mechanism through which presenteeism elucidates the negative impact of conscientiousness on employees’ work engagement has great practical significance. COR theory suggests that individuals tend to protect existing resources and acquire new ones (Hobfoll, 1989, 2001). When individuals gain resources, they are more likely to invest in and acquire additional resources, and when they lose resources, they tend to protect existing resources to prevent further depletion. Conscientious employees are more inclined to work despite their illnesses, especially when their physical health is compromised. Consequently, they may suffer from cumulative health effects, triggering a spiral of resource loss and consequently weakening their willingness to engage in work. Second, they may view conscientiousness as a stable personality trait while overlooking the influence of environmental factors on their expression (Funder, 2006). POS refers to the degree to which employees perceive that an organization values their job contributions and cares about job happiness (Chiaburu et al., 2021), which is a key variable in the psychological connection between employees and organizations. Previous research found that POS moderates outcomes related to stress, individuals, and work. Therefore, this study introduces POS as a moderating variable in the research model.

The main contributions of this study are as follows. First, based on COR theory, this study examines the mediating effect of presenteeism and explores POS as a boundary condition, revealing the negative impact mechanism of conscientiousness on employee work engagement. This result contributes to a dialectical understanding of the influence effects of conscientiousness in the theoretical circle. Second, this study suggests that managers should stimulate employees’ conscientiousness efficacy, enhance employees’ POS, and mitigate the negative impact of conscientiousness to improve employees’ level of work engagement. Third, this research contributes to a deeper understanding of the personality traits (such as conscientiousness) and situational factors (such as POS) that influence work engagement, providing important insights for human resource management (HRM) practices in enterprises.

## 2 Theory and hypothesis

### 2.1 The impact of conscientiousness on employee work engagement

Conscientiousness has been discussed as the most important personality trait in work-related contexts (Zell and Lesick, 2022). Conscientiousness is related to being dependable, achievement-striving, hardworking, persistent, planning-oriented, and task-oriented (Anglim et al., 2020; Barrick et al., 2005). Work engagement refers to the sustained positive state exhibited by individuals at work, reflecting their degree of involvement in the roles they undertake. It specifically encompasses three aspects: vigor, dedication, and absorption (Schaufeli et al., 2006).

As an important personal resource, conscientiousness is a positive predictor of work engagement. First, according to COR theory, individuals tend to acquire and maintain the resources they value. Conscientious employees consider diligence, focus, and adherence to rules as important resources for completing work tasks. They believe that demonstrating diligence, focus, and adherence to rules in their work makes it easier to earn the trust of supervisors and colleagues, thereby gaining their support to help achieve work goals (Zellars et al., 2006). Therefore, to protect (or sustainably acquire) the various resources brought about by conscientiousness, employees typically exert greater effort in their work. Second, the resource investment principle of COR theory advocates that individuals need to invest and develop resources to prevent resource loss. Conscientious employees are achievement-oriented (McCrae and Costa, 1991). To achieve desired performance, conscientious employees are willing to invest in various resources for organizational development. They typically set clear goals for themselves, develop detailed plans for goal attainment, and dedicate a considerable amount of time to achieve their objectives (Barrick et al., 1993). Third, according to the COR theory, individuals tend to invest more resources to protect their existing resources (Hobfoll, 1989). Conscientious employees consider difficulties in pursuing goals as challenges to be solved. To address this challenge, they are likely to participate conscientiously in training programs and apply the knowledge and skills acquired during training to their actual work. They integrate job demands and resources with their own abilities and needs, and obtain necessary work resources, thereby enhancing their work engagement. Chen et al. (2017) indicated that highly conscientious employees are more committed to their work and engage in voluntary helping behaviors.

Therefore, we proposed the following hypothesis:

*H1*: Conscientiousness positively influences work engagement.

### 2.2 The mediating effect of presenteeism

Presenteeism refers to the behavior of coming to work when ill, even though an individual feels unwell and should call in sick (Aronsson and Gustafsson, 2005). It reduces organizational productivity, increases the frequency of employee mistakes, and leads to increased medical insurance costs borne by both the company and employees owing to recurrent or worsening illness. In addition, it negatively affects employees’ families and work environments.

Bierla et al. (2013) found that individuals with a strong sense of conscientiousness are more likely to engage in presenteeism. According to COR theory, individuals’ valuable resources for survival and development include their time and energy. To achieve a sense of meaningfulness in work and fulfill their self-worth, individuals with high conscientiousness tend to invest considerable time and effort in their work to ensure the smooth operation of the organization. At this point, high conscientiousness may become a source of pressure for individual resource loss. Increased expectations of responsibilities and goals compel them to devote more time and energy even when they are sick or in poor health (Funk, 2024).

The COR theory posits that individuals’ work behaviors and attitudes are related to their perceived resources. If the depletion of one resource is not replenished by another resource, individuals may experience negative work outcomes (Halbesleben et al., 2014). Negative experiences quickly deplete resources. And once resource loss occurs, individuals may fall into a loss spiral, further accelerating future resource depletion (Hobfoll, 2001). Under normal conditions, recovery occurs when individuals are no longer confronted with work demands or when stress is reduced (Geurts and Sonnentag, 2006). When individuals become ill, or their health deteriorates, they require resources for recovery. These resources include time for rest and temporary relief from work. However, presenteeism deprives them of the opportunity to recover from illness and reduces their access to recovery resources. Failing to fully recover while still going to work, workers may suffer the cumulative health impairments. In turn, emotional responses toward their work in general may be lessened or become negative, resulting in increased fatigue, tension, and anxiety, which in turn leads to a decrease in employee work capacity (Karanika-Murray et al., 2015). Presenteeism prevents individuals from fully dedicating themselves to work, thereby reducing their work engagement. Côté et al. (2021) pointed out that presenteeism has a significant negative predictive effect on work engagement.

Sun et al. (2015) demonstrated that highly conscientious employees are aware that absenteeism may result in economic losses for the organization, disrupt organizational operations, or harm their own reputation; therefore, they are more likely to come to work while sick. However, this behavior may lead to various negative consequences, such as a decrease in work engagement.

Therefore, we proposed the following hypothesis:

*H2*: Presenteeism mediates the relationship between conscientiousness and work engagement.

### 2.3 Moderating effect of POS

Employees’ workplace behaviors are influenced by the interaction between individual and situational factors, meaning that the relationship between conscientiousness and presenteeism may be actuated or restrained by environmental factors. As a situational factor, POS supplements the emotional resources provided by the organization at the organizational level (Zhao and Xi, 2017). It is the perception of employees regarding whether the organization values their contributions and cares about their well-being (Eisenberger et al., 1986).

Previous research indicated that POS helps reduce employee presenteeism (Jiang et al., 2023; Gerlach et al., 2024). According to COR theory, in the pathway of conscientiousness’s influence on presenteeism, POS can assist employees in overcoming resource scarcity as an external supplementary resource. As the context of resource loss amplifies the value of resource acquisition, resources obtained during resource loss situations have a greater positive potential (Hobfoll et al., 2018). Conscientiousness drives the occurrence of presenteeism, depriving employees of the opportunity to recover from illness and stress and forcing them to deplete their own resources to cope with work. POS provides employees with the opportunity to reduce or eliminate this negative impact. Compared with employees with lower POS, those with higher POS have more frequent and closer interactions with their leaders and colleagues (Wei, 2022). The sense of care and support from supervisors and the organization reminded them to control their behavior and cut losses in time. When they perceive that their physical health is not sufficient to meet job demands or they believe that coming to work while ill will affect productivity, they are more inclined to seek help from their organization or colleagues, ask colleagues to cover their shifts, or swap shifts until they recover enough to meet job demands before returning to their work positions. At this point, the positive relationship between conscientiousness and presenteeism weakened.

Empirical research suggests that POS can foster positive psychological states among employees, enhance their confidence and motivation to overcome difficult periods, and reduce the negative emotions caused by adverse effects (Eisenberger et al., 2001). In the pathway of presenteeism’s impact on work engagement, according to the Job Demands-Resources model, job resources not only lead to high work engagement, low cynicism, and high job performance but also have a mitigating effect on the health damage caused by job demands (Bakker and Demerouti, 2007). Compared with employees with lower POS, those with higher POS believe that they have received sufficient resources from the organization (Melchiorre et al., 2013). These resources can buffer the depletion caused by negative factors, mitigate the impact of job demands on work pressure (Bakker et al., 2005; Viswesvaran et al., 1999), and help employees better cope with the pressure of working while ill, thereby increasing their work engagement.

Therefore, we proposed the following hypotheses:

*H3*: POS moderates the relationship between conscientiousness and presenteeism such that this relationship weakens as POS increases.

*H4*: POS moderates the relationship between presenteeism and work engagement such that this relationship weakens as POS increases.

In line with Hypotheses 2, 3 and 4, this study proposes a moderated mediation model. POS weakens the positive impact of conscientiousness on presenteeism and strengthens the negative impact of presenteeism on work engagement, while presenteeism plays a mediating role in the relationship between conscientiousness and work engagement. Therefore, we proposed the following hypotheses:

*H5*: POS moderates the indirect effect of conscientiousness on work engagement through presenteeism.

**Figure 1:**
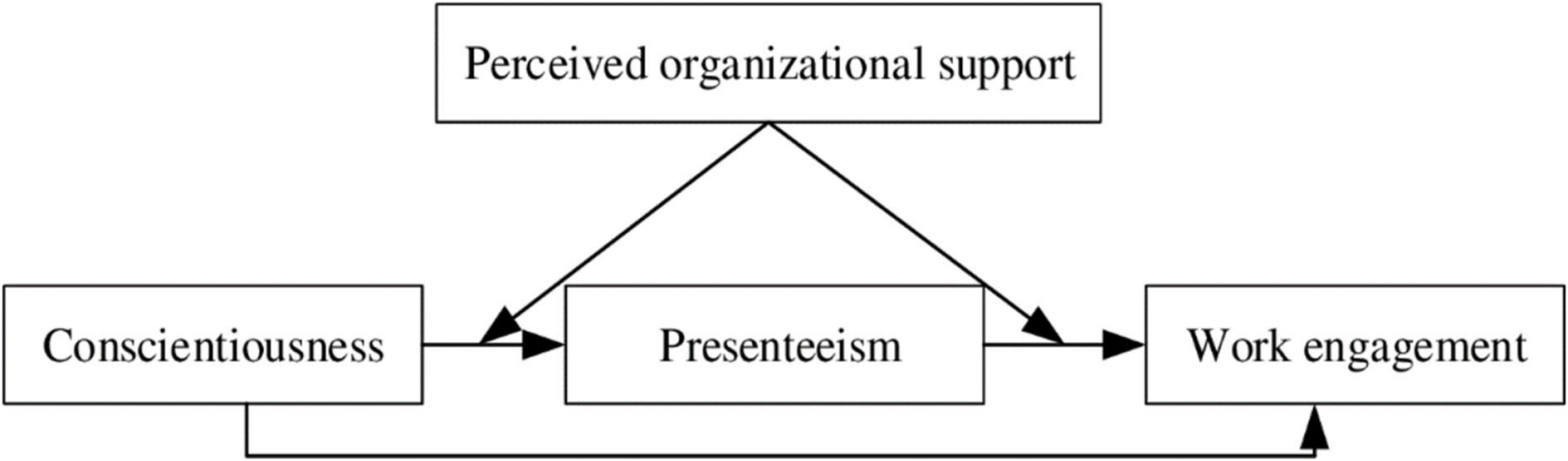
Theoretical model.

The theoretical model of this study is illustrated in Figure 1.

## 3 Methods

### 3.1 Participants and procedures

The sample for this study was drawn from 10 enterprises located in four provinces of China: Jiangsu, Zhejiang, Henan, and Liaoning. To dispel respondents’ concerns about the survey, the research team contacted the responsible persons or HRM departments of the enterprises to clarify the purpose, research questions, and subjects of the survey before the survey occurred. They assured all respondents that their answers would be used solely for academic research purposes, and guaranteed the confidentiality of personal privacy. To reduce the common method bias, data collection for this study was conducted at two time points. At the first time point, employees’ conscientiousness, POS, and demographic variables (gender, age, education level, and organizational tenure) were measured. Presenteeism and work engagement were measured at the second time point (1 month later). A total of 480 questionnaires were distributed in the first phase, with 438 returned; 480 questionnaires were distributed in the second phase, with 425 returned. After matching the questionnaires according to pre-assigned numbers and excluding invalid questionnaires due to regular pattern responses, multiple selections, and so on, 376 valid questionnaires were paired.

Of the valid questionnaires, 238 were from females, which accounted for 63.29% of the sample. The majority of respondents were within the age ranges of 31–40 years (36.97%) and 41–50 years (27.93%). Those with a bachelor’s degree or higher accounted for 68.35% of the sample. Most respondents had 1–10 years of work experience and 11–20 years of work experience, accounting for 38.83 and 31.91% of the sample, respectively. Regarding education, most participants held a bachelor’s degree (68.35%). In terms of organizational tenure, the primary ranges were 1–10 years (38.83%) and 11–20 years (31.91%).

### 3.2 Measures

All the measurement scales used in this study were mature scales from domestic and international sources. Except for the presenteeism questionnaire, all the other questionnaires were measured using a Likert 5-point scale, as follows:

#### 3.2.1 Conscientiousness

Conscientiousness was measured using a 12-item scale developed by McCrae and Costa (1991). Sample items include “I can effectively manage my time to ensure that various tasks are completed on time”.

#### 3.2.2 Presenteeism

We used Lu et al. (2013) two-item scale to measure presenteeism. Some sample items are “Even though you feel unwell, you still force yourself to go to work” and “Even though you have physical symptoms such as headache or backache, you still force yourself to go to work.” We adopted a four-point Likert scale ranging from “1 = Never” to “4 = More than 5 times.”

#### 3.2.3 Work engagement

We used Schaufeli et al. (2006) 17-item scale to measure work engagement. A sample item is “I am absorbed in my work”.

#### 3.2.4 Perceived organizational support

We used Eisenberger et al. (1997) 17-item scale to measure POS. A sample item is “Help is available from the organization when I have a problem”.

#### 3.2.5 Control variables

To avoid possible confounding effects, we controlled for sex, age, education, and organizational tenure in all analyses.

### 3.3 Ethical approval

Ethical review and approval was not required for this study on human participants in accordance with the local legislation and institutional requirements. The patients/participants provided their written informed consent to participate in this study.

## 4 Data analysis and results

### 4.1 Confirmatory factor analysis and common method bias control

To assess the discriminant validity of the variables involved in this study, the Amos 23.0 software package was used to conduct a confirmatory factor analysis of the four main research variables: conscientiousness, presenteeism, work engagement, and POS. The results indicated that the four-factor model provided the best fit. All fit indices were above acceptable levels (*χ*^2^/df = 1.95, CFI = 0.93, TLI = 0.92, RMSEA = 0.05, SRMR = 0.05), and the four-factor model demonstrated significantly better fit than other nested models. Therefore, the discriminant validity of the four main constructs in this study was good. Additionally, the Harman’s single-factor analysis showed that the variance explained by the first extracted factor was 23.59%, which is less than the empirical threshold of 40%. Hence, it can be concluded that common method bias in this study was relatively low.

### 4.2 Descriptive statistics and correlation analysis

Table 1 reports the descriptive statistics and correlation coefficients of the research variables. Conscientiousness is significantly positively correlated with work engagement (*r* = 0.42, *p* < 0.001); conscientiousness is significantly positively correlated with presenteeism (*r* = 0.39, *p* < 0.01); presenteeism is significantly negatively correlated with work engagement (*r* = −0.32, *p* < 0.01). These results provide preliminary evidence for further hypothesis testing.

**Table 1:** Correlation coefficient and descriptive statistical analysis of variables.

Variables	1	2	3	4	5	6	7	8
1. Gender	1							
2. Age	−0.08	1						
3. Education Level	−0.02	0.14	1					
4. Organizational tenure	0.16	0.42^**^	0.07	1				
5. Conscientiousness	0.02	−0.11	0.03	0.18	1			
6. Presenteeism	0.11	0.19	−0.04	0.07	0.39^**^	1		
7. Work Engagement	0.13	0.05	0.02	0.21	0.42^***^	−0.32^**^	1	
8. POS	0.17	−0.14	−0.07	0.14	0.21*	−0.24^*^	0.29^**^	1
M	1.63	2.53	2.48	2.75	3.35	3.33	3.42	3.53
SD	0.50	0.84	0.65	0.89	0.37	0.62	0.46	0.52

*N* = 376, ^***^p<0.001^**^; *p*<0.01; ^*^p<0.05, two-tailed test.

### 4.3 The mediating effect of presenteeism

To test the mediating effect of presenteeism on the relationship between conscientiousness and work engagement, Model 4 of the PROCESS was employed. The results (Table 2) indicate that after controlling for the variables, conscientiousness has a significant positive impact on work engagement [*β* = 0.44, 95% CI (0.38, 0.59)]. Thus, H1 is supported. In addition, conscientiousness has a significant positive impact on presenteeism [*β* = 0.25, 95% CI (0.18, 0.30)], and when conscientiousness and presenteeism simultaneously predicted work engagement, the influence coefficient for the former was 0.48, 95% CI [0.41, 0.64], while that for the latter was −0.17, 95% CI [−0.29, −0.11]. These results indicate that presenteeism partially mediates the relationship between conscientiousness and work engagement, supporting H2.

**Table 2:** Mediation effect test of presenteeism.

Variables	Outcome variable: Presenteeism		Outcome variable: Work Engagement		Outcome variable: Work Engagement	
	*β*	*CI*	*β*	*CI*	*β*	*CI*
Gender	0.08	[−0.06,0.14]	0.11	[−0.04,0.18]	0.08	[−0.05,0.14]
Age	0.10	[−0.05,0.17]	0.02	[−0.03,0.04]	0.01	[−0.02,0.03]
Education Level	−0.02	[−0.01,0.04]	0.01	[−0.05,0.02]	0.01	[−0.03,0.03]
Organizational tenure	0.07	[−0.08,0.12]	0.13	[−0.01,0.21]	0.11	[−0.01,0.20]
Conscientiousness	0.25	[0.18,0.30]	0.44	[0.38,0.59]	0.48	[0.41,0.64]
Presenteeism					−0.17	[−0.29,-0.11]
*R^2^*	0.06 12.21^**^		0.20		0.26	
*F* value			73.24^***^		83.97^*^	

*N* = 376; ^***^p<0.001^**^; ^**^p<0.01; ^*^p<0.05.

### 4.4 Moderation effects and moderated mediation test

To test the moderating effect of organizational support, Model 58 from PROCESS was employed in the analysis. The specific data are presented in Table 3.

**Table 3:** Moderation effect test of POS.

Variables	Outcome variable: Presenteeism		Outcome variable: Work engagement	
	*β*	95%*CI*	*β*	95%*CI*
Gender	0.08	[−0.09,0.20]	0.12	[−0.01,0.25]
Age	0.11	[−0.02,0.26]	0.01	[−0.02,0.10]
Education Level	−0.00	[−0.03,0.10]	0.02	[−0.02,0.11]
Organizational tenure	0.09	[−0.05,0.31]	0.15	[−0.01,0.33]
Conscientiousness	0.16	[0.08,0.34]		
Presenteeism			−0.46	[−0.58, -0.29]
POS	−0.41	[−0.54,-0.23]	0.24	[0.13,0.22]
Conscientiousness *POS	−0.10	[−0.15, -0.04]		
Presenteeism *POS			0.18	[0.06, 0.40]
*R^2^*	0.23		0.73	
*F* value	19.18^**^		64.26^**^	

*N* = 376; ^***^p<0.001^**^; ^**^p<0.01; ^*^p<0.05.

Table 3 indicates that the interactive effects of conscientiousness and POS on presenteeism were negative and significant [*β* = −0.10, 95% CI (−0.15, −0.04)]. The absence of zero in CI indicates that H3 is supported. Additionally, the interactive effects of presenteeism and POS on work engagement were positive and significant [*β* = 0.18, 95% CI (0.06, 0.17)]. The absence of zero in CI indicates support for H4.

**Figure 2:**
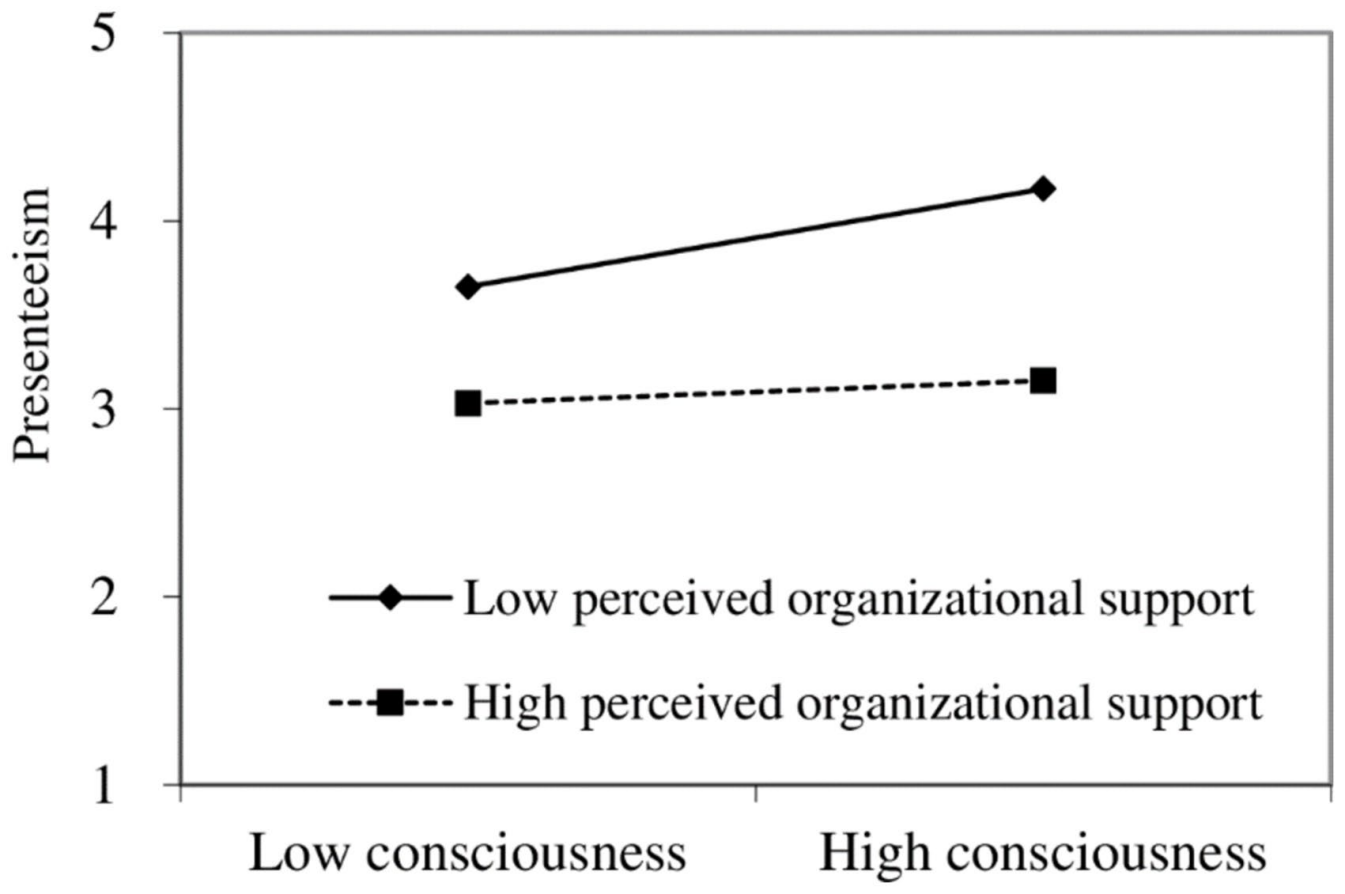
Moderating role of POS in the relationship between consciousness and presenteeism.

To illustrate the moderating effect of POS better, a simple slope analysis was conducted. Figure 2 shows that under low POS (M-1SD), conscientiousness positively predicts presenteeism significantly [*β* = 0.20, 95% CI (0.10, 0.21)], whereas under high POS (M + 1SD), conscientiousness does not significantly predict presenteeism [*β* = 0.02, 95% CI (−0.03, 0.03)].

**Figure 3:**
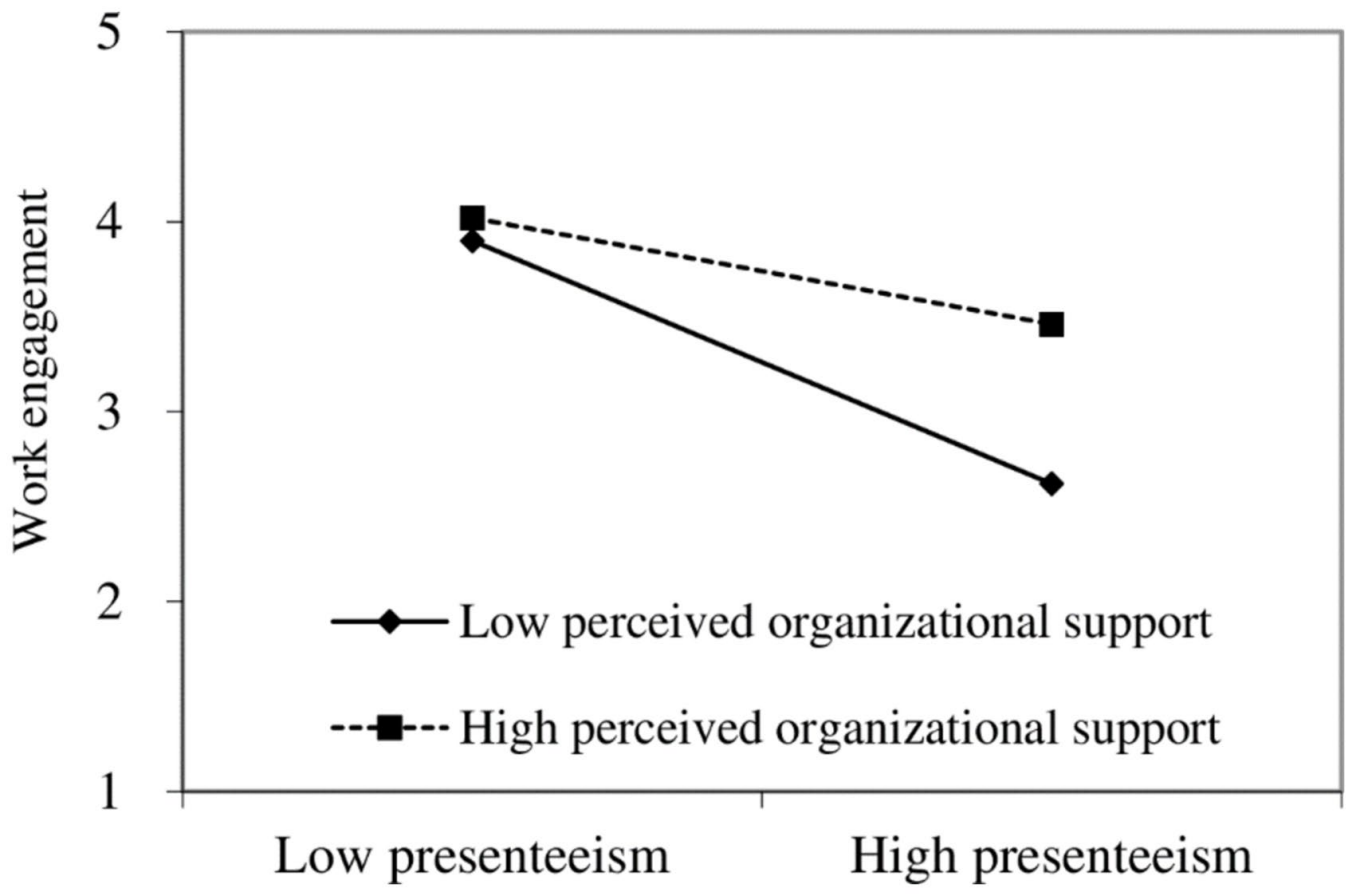
Moderating role of POS in the relationship between presenteeism and work engagement.

Figure 3 indicates that under low POS (M-1SD), presenteeism negatively predicts work engagement significantly [*β* = −0.32, 95% CI (−0.35, −0.20)]; and under high POS (M + 1SD), presenteeism also significantly predicts work engagement [*β* = −0.13, 95% CI (−0.18, −0.09)], but to a lesser extent.

In order to analyze the mediation effect of moderated mediation models, this study conducted a simple slope analysis on the mediation effect (Fang et al., 2014). Using the mean ± 1SD as the standard, distinguish the high and low levels of moderating variables, and perform a simple slope analysis on the mediating effect. The results (Table 4) showed that under the average POS(M), the mediating effect of presenteeism on conscientiousness and work engagement was significant [mediation effect size *β* = −0.12, 95% CI (−0.20, −0.11)]; Under low POS (M-1SD), the mediating effect of presenteeism on conscientiousness and work engagement was also significant [mediation effect size *β* = −0.17, 95% CI (−0.26, −0.13)], but the mediating effect size of presenteeism has increased by about 41.67% compared to when the POS is the mean; Under high POS (M + 1SD), the mediating effect of presenteeism on conscientiousness and work engagement was also significant [mediation effect size *β* = −0.09,95% CI (−0.17, −0.04)], but the mediating effect size of presenteeism has decreased by 25% compared to the mediating effect size when POS was the mean. Moreover, there was a significant difference in the mediating effect size between high and low POS [*β* = 0.08, 95% CI (0.03, 0.18)]. These results indicate that POS moderates the indirect effect of conscientiousness on work engagement through presenteeism, whereas the mediated relationship is weakened when employees exhibit a higher POS. Therefore, H5 was supported.

**Table 4:** The results of simple slope analysis of mediation effect.

	*β*	Estimate(SE)	95% CI
Low POS(M-1SD)	−0.17	0.14	[−0.26, −0.13]
Average POS(M)	−0.12	0.13	[−0.20, −0.11]
High POS (M + 1SD)	−0.09	0.10	[−0.17, −0.04]
Estimate difference	0.08	0.04	[0.03, 0.18]

## 5 Discussion

Based on COR theory in the Chinese context, this study explored the impact of conscientiousness on employees’ work engagement and examined the mediating effect of presenteeism and the moderating effect of POS. Results showed that conscientiousness significantly and positively predicted work engagement. Presenteeism partially mediated the relationship between conscientiousness and work engagement. POS negatively moderated the relationship between conscientiousness and presenteeism, and positively moderated the relationship between presenteeism and work engagement. Consciousness has a negative indirect effect on work engagement via presenteeism, and the indirect effect is weaker when POS is higher than when it is lower.

### 5.1 Theoretical contributions

First, we are aware of very little about how conscientiousness leads to negative outcomes (Liu et al., 2022). This study investigated the negative impact of conscientiousness on employee work engagement in terms of the negative aspect of presenteeism. The results indicate that although conscientiousness may be associated with many positive outcomes, it can also be costly (e.g., increased presenteeism). By investigating the negative effects of conscientiousness, this study compensates for the shortcomings in research on the influence of conscientiousness, responds to the academic community’s call for exploration of situational variables affecting individual traits(Judge et al., 2014), and provides insights and references for further investigations into the impact of conscientiousness.

Second, our study extends the literature on work engagement by revealing that individual traits may lead to high presenteeism and low work engagement. Prior studies on individual traits and work engagement focused only on one perspective: how individual traits promote work engagement, such as core self-evaluations and proactive personality (Kleine et al., 2019). Hence, our finding that conscientiousness, a relatively bright trait, can inhibit work engagement suggests a new perspective to explore how individual traits affect work engagement.

Third, POS was introduced as a moderating variable to explore the boundary conditions of conscientiousness in employee work engagement. POS enhances employees’ trust in their supervisors or organizations because of perceived support, understanding, and recognition of their abilities by colleagues and leaders (Chen and Liao, 2006). Accordingly, the study found that the higher the POS of employees, the weaker positive effect of conscientiousness on presenteeism and the negative impact of presenteeism on work engagement, meanwhile, the weaker the mediating effect of presenteeism between conscientiousness and work engagement. This study not only deepens scholars’ understanding of the context in which conscientiousness affects employee work engagement but also provides new insights for future research on the boundary conditions of other personality traits influencing employee behavior and work outcomes.

### 5.2 Practical implications

First, the results should prompt managers of the potentially negative impacts of conscientiousness on work engagement. Organizations must take a dialectical view of the mechanisms by which conscientiousness affects employees’ work engagement. Attention should be paid to the influence of conscientiousness on employees’ presenteeism, reminding employees to be aware of resource depletion and dynamically managing employees’ conscientiousness.

Second, resource support should been provided to employees to improve their POS. This empirical research demonstrates that POS not only effectively mitigates the positive impact of conscientiousness on presenteeism, but also alleviates the negative impact of presenteeism on work engagement. Therefore, managers should focus on caring for organizational members, valuing their life and psychological needs, and providing comprehensive work support from both material and spiritual perspectives. Constructing a conducive work environment that weakens employee presenteeism can reduce the negative impact of conscientiousness on work engagement.

Third, constructively manage presenteeism to improve employees’ work engagement. Managers need to be aware that, whether in the short or long term, sickness presenteeism is harmful to employees’ work engagement and performance. To solve the problem at its root, managers should invest in health promotion programs and work-oriented interventions, encouraging sick employees to fully utilize the organization’s sick leave regulations and arrange their workload reasonably.

### 5.3 Limitations and future research

First, questionnaires were administered over different periods. Although the homology variance can be controlled, it still exists objectively. Subsequent research could employ multiple methods such as case studies and experiments to cross-validate the robustness of our conclusions. Second, this study verified that employee presenteeism plays a partial mediating role between conscientiousness and employee work engagement. Subsequent research could explore other mediating effects from different perspectives (e.g., individual motivation and self-efficacy). Third, Gelfand et al. (2011) noted that cultural tightness–looseness varies widely across the world. It plays a crucial role in organizational development, influencing individuals’ information and cognitive processes, and changing the perception of psychology and behavior in organizations (Song et al., 2023). This study was conducted in China, a representative country of a tight culture (have many social norms and a low tolerance of deviant behavior). Future research should consider expanding this research to other countries with loose cultures (have weak social norms and a high tolerance of deviant behavior) to enhance sample diversity and improve the generalizability of the research results. Finally, this study explores the moderating role of POS from the perspective of employees’ perceptions. Future research could shift the focus to managers’ perspectives and explore other moderating factors such as humor-oriented and inclusive leadership, which provide positive emotional resources. This approach could comprehensively clarify the relationship between conscientiousness and work engagement, and offer deeper theoretical insights and practical guidance.

## Data Availability

The raw data supporting the conclusions of this article will be made available by the authors, without undue reservation.
